# Artificial Intelligence in Delirium Care: Predicting, Detecting, and Preventing Delirium in Older Hospitalized Adults

**DOI:** 10.7759/cureus.116995

**Published:** 2026-09-27

**Authors:** Ayra Khan, Abdullah Khan

**Affiliations:** 1 Medicine, Independent Research, NHS Foundation Trust, Surrey, GBR; 2 Paediatrics, Guy's and St Thomas' NHS Foundation Trust, London, GBR

**Keywords:** artificial intelligence, delirium, dementia, frailty, machine learning, natural language processing, older adults

## Abstract

Delirium is a common and serious complication of hospitalisation in older adults, but it is frequently missed because symptoms fluctuate, hypoactive presentations are subtle, and dementia complicates recognition of acute change. Artificial intelligence (AI), including machine learning, natural language processing (NLP), deep learning, and large language models, may support earlier risk stratification, enhanced surveillance, documentation review, and delivery of personalised prevention. This narrative review examines current evidence for AI in predicting, detecting, and preventing delirium in older hospitalised adults. It considers model development, external and temporal validation, calibration, clinical utility, workflow integration, frailty, dementia, multilingual documentation, explainability, fairness, privacy, and accountability. Existing studies show promising discrimination in retrospective datasets, and NLP can identify delirium-related information that is absent from diagnostic codes. However, performance often declines during external validation, calibration is inconsistently reported, and few systems have been tested prospectively in routine care. Established multicomponent non-pharmacological interventions remain the strongest evidence-based approach to prevention, while evidence that AI-triggered recommendations improve patient outcomes is limited. Future research should therefore move beyond technical accuracy toward prospective, multicentre, human-in-the-loop evaluations that measure alert burden, clinician response, intervention fidelity, patient-centred outcomes, and subgroup equity.

## Introduction and background

Delirium, the clinical burden

Delirium is an acute neuropsychiatric syndrome characterised by disturbances in attention, awareness, cognition, and arousal. It is particularly common among hospitalised older adults and is associated with prolonged hospitalisation, functional decline, cognitive deterioration, mortality, and increased healthcare use. An interdisciplinary European statement reports that delirium affects approximately one in five hospitalised older patients, with substantially higher prevalence in intensive care settings, while remaining undetected in many cases [1].

In this review, delirium prediction refers to estimating a patient’s future risk of developing delirium before it is clinically recognised. Delirium detection refers to identifying delirium that may already be present but has not been documented or formally diagnosed. Prevention refers to using risk or detection information to initiate evidence-based interventions, such as orientation, hydration, sensory support, sleep protection, mobility, medication review and family engagement. These applications are related but should not be treated as equivalent outcomes.

Frailty and dementia increase vulnerability to delirium. A systematic review and meta-analysis estimated that frailty was present in approximately 34% of hospitalised older adults and was associated with a 66% higher risk of delirium [2]. Dementia is also one of the strongest established risk factors for incident delirium in older medical inpatients [3]. Delirium is clinically important not only because of its immediate consequences but also because it may represent a marker of acute physiological stress and reduced cerebral reserve. The severity, duration, and motor subtype of delirium may influence subsequent mortality, functional decline, and cognitive outcomes [4].

Challenges in recognition of delirium

Recognition is difficult because delirium is heterogeneous and fluctuates over time. Hyperactive delirium is more visible, whereas hypoactive delirium may present as withdrawal, reduced activity, drowsiness, or reduced communication. In an emergency department study of older adults, 76% of delirium cases were not recognised by emergency physicians, and most cases missed in the emergency department remained unrecognised at hospital admission [5]. The diagnostic challenge is greater in people with dementia, communication difficulties, sensory impairment, or pre-existing functional limitations. The clinician must distinguish acute change from chronic cognitive impairment and obtain information about baseline cognition and behaviour. This often requires family involvement, review of previous records, repeated assessments, and interpretation of subtle changes over time.

Validated instruments such as the Confusion Assessment Method (CAM), the Confusion Assessment Method for the Intensive Care Unit (CAM-ICU), and the 4 ‘A’s Test (4AT) support systematic assessment, but their effectiveness depends on regular use, staff training, and accurate documentation. The 4 ‘A’s Test is a brief screening instrument designed to identify possible delirium and cognitive impairment. The 4AT has demonstrated useful diagnostic performance in acute medical settings, although no screening tool removes the need for clinical judgement [6]. The Confusion Assessment Method (CAM) is a bedside diagnostic algorithm based on acute onset or fluctuating course, inattention, disorganised thinking and altered level of consciousness. The CAM for the Intensive Care Unit adapts this approach for critically ill patients, including those who may be unable to communicate verbally. These tools support clinical assessment but are not interchangeable with diagnostic criteria, electronic health-record codes or natural-language-processing outputs.

Machine-learning models identify statistical patterns in clinical data, natural-language-processing systems analyse free-text documentation, deep-learning models learn complex representations from large datasets, and large language models generate or summarise text but require clinical supervision because fluent output does not guarantee factual accuracy or safe reasoning.

## Review

The primary PubMed/MEDLINE search was conducted in August 2026 using terms for delirium, artificial intelligence and older adults (Table 1). Supplementary targeted searches examined prediction, detection, natural-language processing, prevention, frailty, dementia and large language models. Because the review was narrative rather than systematic, targeted search yields were not added to the primary search yield because substantial overlap was expected. Retrieved records were reviewed for direct clinical relevance to delirium care in older hospitalised adults, and representative primary studies, validation studies, systematic reviews and implementation reports were selected for narrative synthesis. No formal risk-of-bias assessment, duplicate-screening log or meta-analysis was undertaken because the studies differed substantially in population, setting, delirium definition, prediction window, reference standard, validation strategy and reported performance metrics. Confidence intervals were reported when available in the original studies. The review did not involve new statistical analysis.

**Table 1:** The primary PubMed/MEDLINE search was conducted in August 2026 without a publication-date restriction. The targeted searches were supplementary and overlapped substantially with the primary search, so the component yields should not be summed. The records were used to identify representative primary studies, validation studies, reviews and implementation reports for narrative synthesis. Because this is a narrative rather than systematic review, the yields do not represent a deduplicated systematic-review record set.

Search component	Main concepts	Search terms	Records
Primary structured search	Delirium AND artificial intelligence AND older adults	Delirium or acute confusion; artificial intelligence, machine learning, deep learning, natural language processing, large language models, generative AI, computer vision or predictive models; older adults, elderly, geriatric, aged or older people	208
Prediction	Delirium AND artificial intelligence AND prediction	Prediction-focused combination of the primary search concepts	44
Machine learning	Delirium AND machine learning AND older adults or elderly	Machine-learning-focused combination of the primary search concepts	72
Natural language processing	Delirium AND natural language processing AND older adults or elderly	NLP-focused combination of the primary search concepts	5
Detection	Delirium AND artificial intelligence AND detection	Detection-focused combination of the primary search concepts	26
Prevention	Delirium AND artificial intelligence AND prevention	Prevention-focused combination of the primary search concepts	21
Frailty	Delirium AND artificial intelligence AND frailty	Frailty-focused combination of the primary search concepts	10
Dementia	Delirium AND artificial intelligence AND dementia	Dementia-focused combination of the primary search concepts	14
Large language models	Delirium AND large language models or generative AI	LLM-focused combination of the primary search concepts	14

Eligibility criteria

Studies were considered if they involved hospitalised adults aged 65 years or older, or if their findings were directly relevant to the care of hospitalised older adults. Studies involving mixed-age populations were considered when the clinical setting, outcome, or application was relevant to geriatric care. Eligible artificial intelligence (AI) approaches included machine learning, deep learning, natural language processing (NLP), large language models (LLMs), computer vision, predictive modelling, and artificial intelligence-enabled electronic health record (EHR) analysis. Studies addressing prediction, detection, diagnosis, monitoring, prevention, management, or clinical decision support were considered (Table 2).

**Table 2:** Inclusion and exclusion criteria.

Domain	Inclusion criteria	Exclusion criteria
Population	Hospitalised adults aged 65 years or older; mixed-age studies directly relevant to older inpatients; medical, surgical, emergency, intensive care, and geriatric settings	Paediatric populations; community-only studies without direct hospital relevance; studies in which older adults could not be meaningfully evaluated
Clinical topic	Delirium prediction, detection, diagnosis, monitoring, prevention, management, or clinical decision support	Studies addressing dementia, falls, cognitive impairment, or mortality without a meaningful delirium component
AI method	Artificial intelligence, machine learning, deep learning, NLP, LLMs, computer vision, or predictive modelling	Conventional statistical studies without an AI or predictive-modelling component, unless used as a comparator
Data source	Electronic health records, clinical notes, physiological data, EEG, imaging, medication data, laboratory data, or multimodal hospital data	Purely theoretical or technical studies without clinically relevant patient data
Study type	Original studies, validation studies, implementation studies, clinical decision-support studies, systematic reviews, scoping reviews, and relevant narrative reviews	Editorials, commentaries, protocols, duplicate publications, conference abstracts without sufficient results, and studies lacking clinically interpretable outcomes
Outcomes	Discrimination, calibration, sensitivity, specificity, predictive values, clinical utility, workflow impact, delirium incidence, severity, duration, length of stay, mortality, function, or safety	Studies reporting algorithm performance without sufficient information about the population, outcome, or validation strategy
Language	No language filter was applied in the PubMed/MEDLINE search	Studies for which no interpretable abstract or reliable full-text information was available

Search yield and narrative synthesis

The primary PubMed/MEDLINE search returned 208 records. The targeted searches were supplementary and overlapped substantially with the primary search. Their yields are shown in Table 3. The records were used to identify representative primary studies, validation studies, reviews, and implementation reports for narrative synthesis. The search yields represent records returned by individual search components and should not be interpreted as unique studies or as the number of included studies. The targeted search yields were supplementary and overlapped with the primary search. The final reference list represents selected sources used for narrative synthesis rather than a formally screened systematic-review cohort.

**Table 3:** Search yield.

Search component	Main concepts	Number of records
Primary structured search	Delirium, AI-related terms, and older-adult terms	208
Prediction	Delirium, artificial intelligence, and prediction	44
Machine learning	Delirium, machine learning, and older adults or elderly	72
Natural language processing	Delirium, natural language processing, and older adults or elderly	5
Detection	Delirium, artificial intelligence, and detection	26
Prevention	Delirium, artificial intelligence, and prevention	21
Frailty	Delirium, artificial intelligence, and frailty	10
Dementia	Delirium, artificial intelligence, and dementia	14
Large language models	Delirium, large language models, or generative AI	14

Prediction models, validation, transportability, and model complexity

Prediction models aim to identify patients likely to develop delirium before clinical recognition. This application is potentially valuable because it may allow clinicians to begin prevention before symptoms become established. Early work demonstrated that electronic health record data could estimate delirium risk among newly hospitalised patients. In a study of more than 18,000 hospital admissions, a gradient-boosting model achieved an area under the receiver operating characteristic curve of 0.855, compared with 0.678 for the conventional AWOL risk score. However, the positive predictive value remained modest at a specificity threshold of 90%, illustrating the difference between discrimination and clinical efficiency [7].

A random-forest algorithm implemented in a clinical workflow generated more than 5000 prospective predictions and achieved an area under the receiver operating characteristic curve of 0.86. However, calibration was poor during clinical use. This finding demonstrates that a model may retain discrimination while producing risk estimates that do not correspond reliably to observed probabilities [8]. More recent models have incorporated temporal electronic health record data. A model combining LightGBM with a long short-term memory network analysed more than 331,000 confusion assessment method (CAM) assessments from over 34,000 patients and achieved an area under the curve of 0.952 in a held-out dataset. Temporal modelling may be useful because delirium risk changes with illness severity, sedation, mobility, medication exposure, and physiological instability [9]. A scoping review of 39 inpatient delirium prediction studies reported a median area under the receiver operating characteristic curve of 0.85 for in-silico performance. However, only six studies reported external validation, and the median area under the curve fell to 0.75 in externally validated models. Only three studies evaluated models within clinical workflows [10]. The DELIrium risK Tool illustrates the potential value of parsimonious models based on routinely available variables. The model included age, clinical department, dementia, neurological impairment, catheterisation, laboratory variables, polypharmacy, and anticholinergic burden. External validation produced an area under the curve of 0.795 [11].

The supporting SURgery with GEriatric Co-Management and Artificial Intelligence (SURGE-Ahead) project incorporated pre-existing dementia, clinical frailty, multi-morbidity, polypharmacy, falls, cognitive testing, and peri-operative variables. The model achieved an area under the curve of 0.76 in cross-centre validation. Its inclusion of frailty and cognitive features is relevant because these characteristics may influence both biological susceptibility and the feasibility of preventive interventions [12]. The evidence does not establish that complex algorithms are consistently superior to simpler approaches. In a temporal validation study of postoperative delirium models, XGBoost did not significantly outperform least absolute shrinkage and selection operator (LASSO) regression, while a parsimonious logistic model achieved comparable predictive performance [13].

Earlier systematic evidence provides an important benchmark for interpreting newer AI models. A systematic review of delirium prediction models in older inpatients identified 23 models, of which 14 had external validation, and three had internal validation. Reported discrimination varied widely, with area-under-the-curve values ranging from 0.52 to 0.94, and the review identified inconsistent delirium ascertainment and incomplete reporting of model performance [14]. Automated prediction has also been evaluated outside purely retrospective development datasets. The DEMO model was externally evaluated in older hospital patients and showed sensitivity of 87.1%, 84.2%, and 82.7% for delirium within one, three, and five days, respectively, with corresponding specificities of 77.9%, 81.5%, and 84.5%. The investigators nevertheless emphasised the need for prospective validation using in-person delirium confirmation, illustrating that a reasonable validation result does not establish clinical readiness [15].

A further external-hospital evaluation of delirium prediction software reported high discrimination after models were retrained using local data, together with favourable clinician ratings of usefulness and ease of use. Retraining improved local performance, but it also raises an operational question: whether a health system can maintain the technical capacity, governance, and data quality needed for repeated local adaptation [16]. Recent work using critical-care databases demonstrates both the potential and the limitations of cross-dataset validation. In an older intensive-care cohort, an extreme-gradient-boosting model achieved internal area-under-the-curve values of approximately 0.85, but performance fell to approximately 0.74 to 0.78 in an external dataset. This decline is clinically important because it shows how differences in case mix, measurement practices, treatment patterns, and documentation can reduce transportability [17].

The SURGE-Ahead postoperative delirium algorithm provides another example of external testing. In a prospective validation cohort, a model using preoperative clinical and demographic features achieved an area under the receiver operating characteristic curve of 0.86 and a Brier score of 0.14. Such results are encouraging, but the study population was relatively small and surgical, so generalisability to medical wards, emergency departments, and patients with advanced dementia remains uncertain [18]. Model complexity should be justified by clinical benefit rather than performance alone. An interpretable intensive-care model using Shapley Additive Explanations (SHAP)-based explanations identified Glasgow Coma Scale, mechanical ventilation, and sedation among the most influential features, while reporting discrimination, calibration, and decision-curve measures. Explanations are potentially useful when they point to modifiable risks, but feature importance should not be interpreted as proof that changing a feature will prevent delirium [19].

Patients with dementia require specific evaluation rather than simple inclusion as a covariate. A prospective model developed in older adults with dementia reported an area under the receiver operating characteristic curve of 0.93 and used SHAP to identify clinical and medication-related predictors. The apparent performance is promising, but the model requires validation in other dementia populations, care settings, languages, and levels of cognitive and functional impairment before it can be used to guide personalised prevention [20].

Detection models, NLP, digital phenotyping, and multilingual documentation

Detection models attempt to identify delirium that is already present but has not been recognised or documented. This application may be particularly useful for hypoactive delirium and for retrospective research, where diagnostic codes and discharge summaries may underrepresent the true delirium burden. Natural language processing (NLP) can extract delirium-related information from clinical notes. In a cohort of older adults, an NLP algorithm based on the Confusion Assessment Method demonstrated high sensitivity, specificity, and accuracy compared with manual chart review. The NLP approach also identified more delirium cases than diagnostic coding alone [21]. A separate study used clinical notes from multiple hospitals and trained support vector machine, recurrent neural network, and transformer classifiers on more than 200,000 labelled sentences. The transformer model achieved a micro-averaged F1 score (which combines precision and recall) of 0.978 and a positive area under the curve of 0.984 in the reported evaluation. NLP detections showed stronger associations with delirium-related medications, sitter or restraint orders, mortality, and CAM scores than ICD codes [22].

Sentiment-based NLP provides an example of how contextual information may improve classification. In nearly 4000 general medical admissions, a model incorporating sentiment information from diagnostic imaging reports achieved an area under the curve of 0.930, compared with 0.869 for a corresponding model without the NLP feature [23]. NLP can also reveal limitations in clinical documentation. In a large real-world study, NLP identified a higher delirium rate than ICD coding, but incomplete notes, changing documentation practices, and missing records remained potential sources of underestimation [24].

Detection should therefore be regarded as clinical surveillance rather than autonomous diagnosis. An NLP system may identify documentation suggestive of inattention, altered arousal, fluctuating cognition, or behavioural change, but final diagnosis requires clinical assessment, review of baseline cognition, identification of precipitating factors, and consideration of dementia, depression, medication effects, and other mimics. The evidence base for NLP detection has expanded, but its limitations are increasingly visible. A systematic review of NLP techniques for delirium detection identified 13 studies involving more than 450,000 patients. Sensitivity varied substantially across studies, transformer models generally achieved the highest reported discrimination, and more than half of the included studies were judged to have a high risk of bias. Only one study reported external validation, and none evaluated prospective implementation or patient outcomes [25].

NLP may improve ascertainment when conventional coding is incomplete. In a multi-centre study of hospitalised patients, an NLP-CAM algorithm captured 80% of delirium identified by the combined reference approach, compared with 55% for clinician documentation and 43% for nursing documentation. The result supports NLP as a surveillance and research tool, but it does not establish that an algorithm can independently diagnose delirium or replace bedside assessment [26]. Language and documentation context directly affect model portability. A semiautomatic Chinese keyword instrument based on electronic medical records showed area-under-the-curve values of 0.72 to 0.79 across documentation groups when compared with assessment based on the Diagnostic and Statistical Manual of Mental Disorders, Fifth Edition (DSM-5). The variation between physician-only and physician-plus-nursing documentation demonstrates that missing note types can alter apparent performance [27].

In critical-care settings, NLP applied to progress notes has been used to identify delirium-suggestive behavioural disturbances at scale. Analysis of nearly 200,000 progress notes identified a large group of patients with behavioural terms associated with delirium risk. This approach can support epidemiological surveillance, but behavioural language is not equivalent to a formal delirium diagnosis and may be influenced by sedation, mechanical ventilation, neurological illness, and local documentation culture [28]. Simpler language features remain useful. A study of medical records identified a small set of delirium-related words and phrases with positive predictive values ranging from 60% to 100%, with keywords recorded more frequently in nursing notes than physician notes. These findings support hybrid systems that combine transparent keyword rules with statistical models, particularly when clinical teams need to understand why a record was flagged [29]. More recent clinical-notes models have compared expert-defined dictionaries with free-text machine learning. A dictionary-based approach achieved higher discrimination than a free-text approach, and inclusion of negative delirium-associated features improved classification. This suggests that domain knowledge remains valuable even when deep learning is available, because delirium documentation contains both positive descriptions and explicit statements that symptoms are absent [30].

Prevention and AI-enabled intervention

The strongest evidence for delirium prevention remains based on multicomponent non-pharmacological interventions rather than AI. A Cochrane review found moderate-certainty evidence that multicomponent non-pharmacological interventions reduced delirium incidence among hospitalised non-intensive care patients by approximately 43%, although evidence for mortality benefit was absent [31]. Clinical trials of modified Hospital Elder Life Program interventions have demonstrated reductions in delirium. In older adults undergoing abdominal surgery, a programme involving orienting communication, oral and nutritional assistance, and early mobilisation reduced postoperative delirium from 15.1% to 6.6% [32]. A tailored, family-involved programme reduced postoperative delirium from 19.4% to 2.6% and was also associated with better functional outcomes and shorter hospitalisation [33]. These findings define the opportunity for AI. An algorithm could identify high-risk patients and link them to interventions addressing orientation, hydration, sensory impairment, sleep, mobility, medication burden, nutrition, and family engagement. However, risk prediction alone does not prevent delirium. The effectiveness of an AI system depends on whether the alert is received, understood, acted upon, and connected to an intervention delivered with adequate intensity and fidelity. The literature identified for this review contains digital-platform proposals and AI-enabled geriatric decision-support projects, but limited evidence from completed trials in which an AI-generated risk signal directly triggered a delirium-prevention intervention and improved patient outcomes. A proposed digital platform combined Internet of Things data, NLP, and AI to support delirium care, while the SURGE-Ahead programme was designed to provide digital geriatric co-management and continuity-of-care recommendations [34,35].

Linking prediction to effective prevention

AI-enabled prevention should be built on interventions that already have clinical evidence. The landmark multicomponent intervention trial by Inouye and colleagues reduced both the number and duration of delirium episodes in hospitalised older patients, while not significantly changing delirium severity or recurrence. This distinction is important: prevention systems should be designed to reduce incident delirium and preserve function, not merely to increase the number of alerts [36]. The Hospital Elder Life Program provides the most established framework for translating risk information into action. A systematic review and meta-analysis of the program found a significant reduction in delirium incidence, together with reductions in falls and potential cost savings. The intervention addresses cognitive impairment, sleep disruption, immobility, sensory impairment, and dehydration, which are precisely the types of modifiable factors that an AI system could help identify and prioritise [37].

Implementation evidence shows that effectiveness depends on sustainability. A community hospital maintained and expanded the Hospital Elder Life Program over seven years and across six inpatient units by using dedicated staffing, local adaptation, volunteer recruitment, and efficient data collection. These organisational requirements imply that an AI alert without personnel, time, and ownership may increase workload without delivering prevention [38]. Medication review is another possible bridge between prediction and intervention. A large quality-improvement program using formulary changes, technology-assisted medication review, order-set modifications, alerts, and education reduced exposure to several high-risk medications in older adults. AI could support this process by identifying patients at risk and highlighting potentially modifiable medication exposure, but final prescribing decisions must remain clinically supervised [39]. More recent multicomponent programs have combined staff education, repeated screening, non-pharmacological and pharmacological strategies, and specialist consultation. An interrupted time-series evaluation reported reductions in delirium prevalence and falls after program implementation, although effects varied by unit and age group. These findings reinforce the need to evaluate AI as part of a broader care pathway rather than as an isolated prediction tool [40].

The prevention literature also clarifies the limits of technology. Multicomponent non-pharmacological approaches remain the most consistently supported prevention strategy, whereas pharmacological prevention and treatment have not shown comparable certainty. AI may improve targeting, timing, documentation, and adherence, but it cannot substitute for orientation, hydration, sensory support, sleep protection, mobility, family engagement, and medication review [41]. The American Geriatrics Society guideline similarly prioritises primary prevention and recommends multicomponent non-pharmacological measures for older adults at risk of postoperative delirium. An AI system should therefore be assessed by whether it increases delivery of these recommended measures and improves patient outcomes, not only by whether it predicts delirium accurately [42].

Clinical implementation and translation

The principal translational gap is the absence of a clearly validated prediction-to-action pathway. A useful system must answer four questions: which patients should receive an alert, what intervention should the alert trigger, who is responsible for responding, and whether the response improves patient-centred outcomes. A model with high sensitivity may generate too many alerts, creating alert fatigue and reducing staff engagement. A model with high specificity may miss patients who would benefit from prevention. Threshold selection should therefore be based on the resources available for intervention rather than statistical performance alone. Clinical utility should be evaluated using calibration, decision-curve analysis, net benefit, alert burden, response time, intervention fidelity, and patient outcomes. Relevant outcomes include delirium incidence, severity, duration, functional decline, length of stay, institutionalisation, mortality, falls, medication exposure, and patient and family experience. The intervention should be proportionate to the predicted risk and personalised to the patient’s vulnerabilities. For example, a patient with dementia, hearing impairment, immobility, and anticholinergic medication exposure may require a different prevention bundle from a patient whose principal risks are sleep disruption, dehydration, and acute infection.

Evaluation beyond discrimination

A clinically useful delirium system should be evaluated across a sequence of outcomes. Technical evaluation includes discrimination, calibration, sensitivity, specificity, precision, and robustness to missing data. Clinical evaluation should then examine alert frequency, time to review, clinician agreement, changes in preventive-care delivery, medication exposure, intervention fidelity, and unintended consequences. The final stage should measure patient-centred outcomes such as incident delirium, duration, severity, functional decline, length of stay, discharge destination, falls, mortality, and caregiver experience. The target of prediction must also be specified. Predicting delirium within 12 hours, during the next hospital day, or over the whole admission represents different clinical tasks and may require different thresholds. Models should be compared with existing clinical tools and simple statistical baselines, because a complex model that adds little net benefit may be less suitable for routine care than a transparent score that clinicians can use reliably [8,10,13].

Frailty and dementia

Frailty and dementia are not merely demographic variables. They represent clinical states that may affect delirium susceptibility, symptom presentation, treatment response, and the acceptability of digital monitoring. Risk models that omit frailty, dementia, functional status, and cognitive impairment may be less clinically meaningful for geriatric populations. Conversely, models that include these variables must avoid treating frailty or dementia as reasons to limit care or reduce the intensity of preventive interventions [2,3]. Dementia creates a particular diagnostic challenge because the distinction between chronic impairment and acute change requires information about baseline function, behaviour, communication, and daily activity. AI systems that rely exclusively on structured diagnosis codes may fail to capture this distinction. NLP applied to longitudinal records may help identify changes in cognition or behaviour, but such systems must be tested across different documentation styles, languages, and levels of caregiver involvement. A geriatric AI model should therefore incorporate functional status, mobility, sensory impairment, frailty, baseline cognition, medication burden, and caregiver information where available. These variables should support person-centred assessment rather than replace it.

Human-in-the-loop clinical support

AI should function as a clinical support mechanism, not an autonomous diagnostic authority. A human-in-the-loop design allows clinicians to review the prediction, inspect influential variables, assess whether the data are current and plausible, and decide whether an intervention is appropriate. Human-in-the-loop evaluations assess how clinicians interact with AI during real or simulated clinical care, rather than measuring model accuracy alone. In delirium care, they examine whether clinicians understand and trust an AI alert, agree with or override its recommendation, and use it to initiate appropriate interventions addressing cognition, medication exposure, hydration, mobility, sensory impairment and sleep. Relevant outcomes include alert frequency, response time, override rates, intervention fidelity, alert fatigue, unintended consequences, delirium incidence and duration, functional outcomes, and differences between patient groups. Interpretability and workflow integration are essential because many delirium risk factors are clinically modifiable. Predictions identifying sedative exposure, urinary catheterisation, immobility, dehydration, infection or sleep disruption should support an actionable clinical review and appear within existing clinical systems, with clear recommendations, assigned responsibility, documentation support and escalation pathways. Prospective implementation studies should therefore assess clinician acceptance, response rates, calibration, intervention fidelity and patient outcomes, rather than relying solely on retrospective discrimination measures [8].

Ethics, fairness, and governance

AI applications in delirium care raise concerns about privacy, fairness, transparency, accountability, and unequal performance. Older adults with dementia, sensory impairment, limited English proficiency, severe illness, or low digital access may be underrepresented in training datasets or may have systematically different documentation patterns. A review of AI in the healthcare of older people identified concerns regarding bias, inadequate external validation in older populations, privacy, autonomy, insufficient transparency, and safeguarding when systems make errors. Effective interventions for older adults require a person-centred approach and appropriate clinical and technological governance [43]. Documentation bias is particularly relevant to NLP. If clinicians document delirium more frequently in some demographic groups, wards, or hospitals, the algorithm may reproduce differences that reflect documentation practice rather than true differences in disease occurrence. Fairness assessment should therefore include subgroup performance, missingness patterns, calibration, false-negative rates, and the consequences of incorrect predictions. Data drift is another practical concern. Delirium risk changes with surgical practice, medication use, infection patterns, staffing, hospital design, and clinical documentation systems. Models require temporal monitoring and recalibration. External validation should include different hospitals, patient groups, clinical services, and levels of frailty. LLMs create additional risks. A customised delirium-related language model has been evaluated for medication-related questions, but performance varied across datasets and did not consistently reach desired thresholds. This supports the potential of LLMs as research or pharmacy-support tools but does not demonstrate safe autonomous clinical decision-making or improved delirium outcomes [44,45].

Large language models and generative AI

Large language models may assist with summarising records, extracting delirium-related symptoms, supporting medication review, and generating patient or caregiver information. However, fluent language does not guarantee factual accuracy, appropriate uncertainty, or safe clinical reasoning. The available delirium-related evidence remains exploratory, and variable performance across datasets means that these systems should be treated as supervised support tools rather than autonomous diagnostic or prescribing agents [44,45].

Fairness, transparency, and stakeholder governance

Fairness must be considered throughout model development and deployment. Machine-learning systems learn from historical data, so underrepresentation, unequal access, and differences in documentation can produce unequal error rates. Fairness assessment should therefore report subgroup discrimination, calibration, sensitivity, false-negative rates, missingness, and the clinical consequences of incorrect predictions, rather than relying on a single overall performance measure [46]. The choice of outcome or proxy can create hidden bias. A widely cited health-care algorithm produced racial disparities because it used health-care expenditure as a proxy for health need. In delirium care, similar problems could arise if diagnostic codes, use of restraints, medication administration, or frequency of documentation are treated as ground truth without accounting for differences in clinical practice and access to assessment [47]. This is, of course, an analogous example of algorithmic bias in healthcare rather than delirium-specific evidence. Ethical implementation also requires input from professionals and patients. Interviews with clinicians, computer scientists, legal experts, and ethicists concerning an AI-based geriatric dashboard identified concerns about privacy, accuracy, transparency, explainability, unequal access, and redistribution of professional responsibility. These concerns support governance processes that define who can override a model, how errors are reported, and how recommendations are audited [48]. Older adults should be involved in the design and evaluation of systems intended for their care. Focus groups with older adults identified potential benefits such as faster diagnosis and improved coordination, but also concerns about cybersecurity, over-diagnosis, loss of clinical skills, financial influence, and insufficient time for individualised review. Human oversight should therefore be visible in the workflow, not merely stated as a policy principle (Table 4) [49].

**Table 4:** Representative artificial intelligence models and studies in delirium care. AUROC, F1 score, sensitivity, and specificity measure different aspects of model performance and should not be ranked directly across studies. Performance estimates are also affected by differences in population, delirium definition, prediction window, reference standard and validation design.

Domain	Representative study or model	Population or data source	Method and validation	Main finding
Prediction	Wong et al. [7]	More than 18,000 hospital admissions	Gradient-boosting model compared with the AWOL score	AUROC 0.855 versus 0.678 for AWOL; positive predictive value remained modest at a specificity threshold of 90%.
Prediction	Jauk et al. [8]	More than 5,000 prospective predictions	Random-forest model implemented in clinical workflow	AUROC approximately 0.86, but calibration was poor during clinical use.
Prediction	Liu et al. [9]	More than 34,000 patients and 331,000 CAM assessments	LightGBM combined with a long short-term memory network	Held-out AUROC 0.952 using temporal electronic health-record data.
Prediction and validation	Strating et al. [10]	Scoping review of 39 inpatient prediction studies	Review of internal and external validation	Median AUROC 0.85 in in-silico studies and 0.75 in externally validated models; only six studies reported external validation.
Prediction	DELIKT [11]	Older non-intensive-care inpatients	Parsimonious model using routinely available clinical variables	External AUROC 0.795.
Prediction and implementation	SURGE-Ahead and related validation studies [12,18]	Surgical and older inpatient populations	Machine-learning models using frailty, dementia, medication and perioperative variables	Performance was encouraging but requires validation in medical wards, emergency departments and people with advanced dementia.
Detection	Fu et al. and Pagali et al. [21,26]	Older hospitalised adults and clinical documentation	NLP models based on CAM-related documentation	NLP identified more delirium-related cases than diagnostic coding and captured a greater proportion of cases than routine clinician or nursing documentation.
Detection	Ge et al. [22]	More than 200,000 labelled clinical-note sentences	Support-vector machine, recurrent neural network and transformer models	The transformer model achieved an F1 score of 0.978 and an AUROC of 0.984 in the reported evaluation.
Detection and portability	Shankar et al. [25]	Systematic review of 13 NLP studies involving more than 450,000 patients	Review of NLP delirium-detection studies	More than half of the studies had a high risk of bias, only one reported external validation, and none evaluated prospective patient outcomes.

Limitations of the evidence and review

The current evidence has several limitations. Many studies are retrospective, single-centre, dependent on routinely collected documentation, and evaluated against imperfect reference standards. External validation remains uncommon, and prospective studies frequently report process outcomes rather than patient-centred outcomes. Heterogeneity in delirium definitions, assessment frequency, prediction windows, datasets, and performance metrics limits direct comparison between models [14,25]. This article is a narrative review and should not be interpreted as an exhaustive systematic review. The literature was searched and synthesised to provide a clinically oriented overview, but no formal risk-of-bias assessment or meta-analysis was undertaken. The conclusions therefore emphasise patterns, translational gaps, and research priorities rather than pooled estimates of effect.

Future research

Future research should move from retrospective algorithm development to prospective clinical evaluation. Priority areas include multicentre and cross-country external validation; prospective temporal validation and recalibration; inclusion of frailty, dementia, sensory impairment, mobility, and baseline cognition; standardised delirium definitions and repeated reference-standard assessments; transparent reporting of calibration, decision-curve analysis, missing data, and subgroup performance; comparison of complex AI models with simpler statistical models; evaluation of alert burden, clinician acceptance, response time, and intervention fidelity; randomised or stepped-wedge studies testing AI-triggered prevention pathways; patient-centred outcomes, including function, quality of life, institutionalisation, and caregiver outcomes; human-in-the-loop governance with clear accountability for clinical decisions; privacy-preserving data sharing and federated validation; and prospective evaluation of LLMs for documentation support, medication review, and patient or caregiver communication. AI research should distinguish between three separate claims: that a model can predict delirium, that clinicians can use the prediction, and that using the prediction improves outcomes. These claims require different study designs and should not be treated as interchangeable.

## Conclusions

Artificial intelligence may support delirium care in older hospitalised adults by improving risk stratification, surveillance, documentation review and the targeting of established multicomponent prevention strategies. However, current evidence does not show that improved algorithmic prediction or detection consistently improves patient outcomes. External validation, calibration, fairness, workflow integration and clinician response remain important unresolved concerns. AI should therefore augment, rather than replace, bedside assessment and clinical judgement. Future studies should evaluate human-in-the-loop systems prospectively across diverse hospitals and patient groups, including people with frailty, dementia, sensory impairment and functional vulnerability. The key endpoint should be whether an AI-supported care pathway increases delivery of effective prevention and reduces delirium incidence, duration, severity and functional decline, rather than whether the model alone achieves high discrimination.
